# Immunotherapy in the Treatment of Human Solid Tumors: Basic and Translational Aspects

**DOI:** 10.1155/2016/7853028

**Published:** 2016-06-01

**Authors:** D. Olive, B. Savoldo, F. Pastorino, R. Castriconi

**Affiliations:** ^1^Centre de Recherche en Cancerologie de Marseille, Inserm, 13009 Marseille, France; ^2^Aix Marseille Université, 13009 Marseille, France; ^3^Institut Paoli-Calmettes, 13009 Marseille, France; ^4^Lineberger Comprehensive Cancer Center, Department of Pediatrics/Hematology-Oncology, University of North Carolina, Chapel Hill, NC 27599, USA; ^5^Laboratorio di Oncologia, Istituto G. Gaslini, 16147 Genova, Italy; ^6^Dipartimento di Medicina Sperimentale, Università degli Studi di Genova, 16132 Genova, Italy; ^7^Centro di Eccellenza per le Ricerche Biomediche, Università degli Studi di Genova, 16132 Genova, Italy

The limited substantial therapeutic progresses observed in the treatment of solid tumors require concerted efforts to combine our thorough knowledge of the tumor biology with a broader comprehension of the main mechanisms regulating antitumor immune responses. In this context, in the last decade, in parallel to the changeless therapeutic quest for targeting molecules responsible for tumor progression, we have witnessed a growing consensus on the potential strong efficacy of combined immunotherapeutic approaches. Recently, these strategies, which include cancer vaccine developments, strenuously pursued for many years, have mainly focused on breaking critical inhibitory immune checkpoint pathways (i.e., PD1/PD-Ls axes), which limit the tumor aggression by different immune cells including natural killer (NK) and T lymphocytes. The strengthening of such endogenous cytolytic effectors, complemented by favoring their migration toward tumors, and/or the infusion of cells engineered with improved function (i.e., with chimeric antigen receptors) is widely considered in additional therapeutic protocols aimed at obtaining a more durable tumor growth control. This goal may also relate to the type of the hematopoietic stem cell transplantation (HSCT) utilized, if included in the treatment of the malignancy.

The papers featured in this issue provide useful information for these rapidly moving above-mentioned fields. Some of them describe new possible mechanisms promoting tumor resistance to effector cell- (like NK cells) mediated aggression or to chemotherapy in the tumor microenvironment favoring tumor progression. Special interest is directed to the possible role for peritumoral NK cells in shaping tumor epithelial mesenchymal transition (EMT) and aggressiveness. C. Goyvaerts and K. Breckpot provide here a useful and comprehensive summary of the current knowledge on the efficacy of vaccination strategies, which differ in the quality of the dispensed antigens and adjuvants, in the modalities of antigen delivery, and in the targeted dendritic cells (DC) subtypes. J. Dannull and colleagues deepen the reasons for the low immunogenicity of immunotherapy strategies based on the infusion of DC-tumor hybrids alone, without adjuvants, whereas another contribution by C. Sakalar and coworkers shows the immunogenic potential of AMHR2 (anti-Müllerian hormone receptor II) molecule in murine models. The efficacy of different vaccination strategies has also been discussed by M. A. Huang and colleagues in the context of pediatric cancer, especially in terms of antibody- and cellular-based therapeutic approaches. These latest interventions include chimeric antigen receptor- (CAR-) T cell based strategies, targeting different antigens in both solid tumors and B cell leukemia. Along this line, B. Clémenceau and colleagues show* in vitro* the efficacy of CAR-NK92 cells targeting the HER-2 antigen in breast cancer, but discouraging* in vivo* activity in a NOD/SCID/IL-2Rg^−/−^ mouse model, due to an off-target effect of the CH2-CH3 hIgG2 spacer of the CAR construct. By interacting with macrophages at the edge of the tumor, the presence of the spacer in the CAR blocked these engineered cells at the periphery of the engrafted tumors, emphasizing critical issues for therapeutic approaches based on engineered molecules and the need for recruitment of effector cells in the tumor mass to obtain clinical benefits. This last point has been also discussed by C. Cantoni and coworkers who highlight the role of HMGB1 in amplifying the recruitment of NK cells towards tumor, in addition to discussing HMGB1 as a promoter of tumor EMT. HMGB1 also represents the focus of an interesting article provided by V. Pistoia and A. Pezzolo who describe HMGB1 as a factor contributing to tumor resistance in antibody-based immunotherapy. C. Cantoni and coworkers also discuss the complexity of immunosuppressive mechanisms acting in the tumor microenvironment and emphasize a possible role of NK cells in exacerbating tumor aggressiveness from residual cancer cells spared by NK cell mediated killing. This NK-mediated protumoral effect may additionally be caused by the induction/upregulation of ligands (i.e., PD-L1) involved in immune checkpoints. Key immune checkpoints involved in tumorigenesis, and more specifically in glioma genesis, have been clearly discussed by E. S. Kim and colleagues who also provide an overview of the existing preclinical and clinical data, antitumor efficacy, and clinical applications for different checkpoints in glioma. Furthermore, group summarizes results of therapies combining the above-mentioned immunotherapeutic approaches with chemotherapy and radiation. Along this line, a comprehensive review is provided by J. Jacobs and colleagues, specifically focusing their discussion on colorectal cancer.

Overall we have clear indications that the achievement of a long-lasting efficacy in the treatment of cancer patients cannot disregard the best combination of different therapeutic approaches, including HSCT, often planned in the standard of care for different malignancies, such as high-risk neuroblastoma or lymphoma. On this subject, R. Fedele and colleagues provide an exhaustive discussion of the clinical benefit of auto- and allo-HSCT options in relapsed or refractory Hodgkin lymphoma, in parallel exploring results derived from either standard or emerging treatment strategies. Finally, the article provided by R. Ortenberg and colleagues focuses on melanoma and points out the relevance of the timing of therapeutic interventions that may be guided by evaluating appropriate serum tumor biomarkers. In this context, the authors proposed the secreted form of CEACAM1 as a useful tumor biomarker endowed with prognostic role.

*D. Olive* *D. Olive* *B. Savoldo* *B. Savoldo* *F. Pastorino* *F. Pastorino* *R. Castriconi* *R. Castriconi*

